# Effect of salt stress in urban conditions on two *Acer* species with different sensitivity

**DOI:** 10.7717/peerj.10577

**Published:** 2021-01-28

**Authors:** Wojciech Dmuchowski, Aneta Baczewska-Dąbrowska, Dariusz Gozdowski, Paulina Brągoszewska, Barbara Gworek, Irena Suwara, Tadeusz Chojnacki, Adam Jóźwiak, Ewa Swiezewska

**Affiliations:** 1 Institute of Environmental Protection—National Research Institute, Warsaw, Poland; 2 Polish Academy of Sciences Botanical Garden—Center for Conservation of Biological Diversity, Warsaw, Poland; 3 Warsaw University of Life Sciences—SGGW, Warsaw, Poland; 4 Institute of Biochemistry and Biophysics, Polish Academy of Sciences, Warsaw, Poland

**Keywords:** Urban trees, *Acer*, Salt stress, Polyprenols, Deicing, Ionic balance

## Abstract

**Background:**

The benefits of trees in urban areas include the following: an increase in ecosystem health, an increase in human health, the mitigation of the effects of heat and drought at microclimate level, the storage and sequestration of carbon, and a reduction in air pollution and noise. These ecosystem services can be provided only by trees that are in good health. The main cause of salt stress in urban environments is the use of de-icing salts on the streets in winter. Salt stress is a complex process that includes changes in plants on the physiological, histological, cellular and molecular levels, leading to limitations in nutrient uptake, disrupting the ionic balance of trees and resulting in the death of roadside trees. In response to salinity, trees have developed a variety of defence mechanisms that allow them to minimize the effects of stress and maintain homeostasis.

**Methodology:**

The reactions of two species *Acer* species: *A. platanoides* and *A. campestre*, which have different sensitivities to the unfavourable conditions of the urban environments (mainly salt stress), were investigated. The research included two experiments: a field experiment with city trees and a controlled pot experiment with young trees treated with increasing doses of salt. In both experiments, the following were performed: an assessment of the health condition of the trees and the content of macroelements as well as the Cl and Na in leaves and a qualitative and quantitative analysis of polyprenols.

**Results:**

*A. campestre* had a more specific strategy than *A. platanoides* for dealing with Na and Cl, which resulted in undamaged leaves. Under the same conditions, *A. platanoides leaves* contained more Cl and Na and were severely damaged. The disruption of the ion balance due to salt stress was lower in *A. campestre* than in *A. platanoides*. Compared with *A. platanoides*, *A. campestre* synthesized more polyprenols in the field experiment. This ability was acquired during the process of acclimation, because it occurred only in the mature trees in the field experiment and not in the young trees in the pot experiment.

**Conclusions:**

The use of two experimental methods (i.e., the field and pot experiments) allowed for a more complete assessment of tree strategies to mitigate salt stress. *A. campestre* displayed a more specific strategy than *A. platanoides*. This strategy was based on several elements*. A*. *campestre* limited Cl and Na transport to the leaves, which resulted in a lack of damage to those organs. Under the same conditions, *A. platanoides* individuals contained more Cl and Na in their leaves and were seriously damaged. *A. campestre* synthesized larger amounts of polyprenols, which probably have the ability to mitigate salt stress. This ability was acquired during the process of acclimation, because it occurred only in the mature trees in the field experiment and was not observed in the young trees in the pot experiment.

## Introduction

We recently entered the “urban age”. Currently, approximately 50% of humanity lives in urban areas, and by 2030, this fraction will have increased to 60% (Wilkinson et al., 2013). In 2018, the population in 561 cities exceeded 1 million (City Population, 2018). The benefits of trees in urban areas include the following: an increase in ecosystem health (Tzoulas et al., 2007), an increase in human health (Ekkel & De Vries, 2017), storage and sequestration of carbon (Nowak et al., 2013), a reduction in air pollution and noise (Nowak et al., 2013; Tong et al., 2016), and provision of cultural services (Dadvand et al., 2016). The role of trees in regulating the urban microclimate is particularly important and includes the following: mitigation of the effects of heat at the microclimate level, a reduction in the urban heat island effect (Lanza & Stone Jr, 2016), microclimate modification by urban shade trees (Takács et al., 2016), urban stormwater management and removal of water from the soil via transpiration of water (Yang et al., 2015; Scharenbroch, Morgenroth & Maule, 2016; Berland et al., 2017). These ecosystem services can be provided only by trees that are in good health.

In cities, especially on streets and in other paved environments, trees are exposed to a number of stressful elements, e.g., heat, low air humidity, periods of drought, high lime content and soil pH, limited soil volume, and de-icing salt (Paludan-Müller et al., 2002; Pauleit, 2003; Vogt et al., 2017). In many cities in the Northern Hemisphere, soil salinity caused by de-icing streets and sidewalks is an important threat to trees, as in the case in North America (Bassuk & Whitlow, 1985; Munck et al., 2010; Ordóez-Barona et al., 2018), northeastern Asia (Kayama et al., 2003; Li et al., 2014), and Europe (Gibbs & Palmer, 1994; Viskari & Kärenlampi, 2000; Cekstere & Osvalde, 2013). In Poland specifically, de-icing has been recognized as the main cause of death of urban trees (Dmuchowski, Baczewska & Bra̧goszewska, 2011a; Dmuchowski, Brogowski & Baczewska, 2011b). In the centre of Warsaw, the number of street trees, despite additional plantings, decreased by 42% during 1973–2016 (Baczewska, Dmuchowski & Puchalski, 2017).

The effect of stress related to soil salinity on trees are complex and includes increases in osmotic pressure in the soil (osmotic stress), specific toxic ion action (salt stress), and nutrient imbalance (Munns & Tester, 2008; Equiza et al., 2017; Ordóez-Barona et al., 2018). Salinity reduces the levels of phenols, carotenoids, and chlorophyll and increase the level of Na^+^ or Cl^−^ ions in the leaves of trees (Cekstere, Osvalde & Nikodemus, 2009; Marosz, 2009). The disturbance of the ionic balance in the leaves caused by the salinity of the soil significantly worsened the health of trees and can decide their death. Among the many indicators of this balance, the ratio of the sum of the organic acids to the sum of the mineral anions actually characterizes this state (Czerniawska-Kusza, Kusza & Duzynski, 2004; Dmuchowski, Brogowski & Baczewska, 2011b; Devitt et al., 2014). Moreover, salt stress can increase (Munck et al., 2010) or decrease (Sienkiewicz-Paderewska et al., 2017) susceptibility to pathogens. Plants have many defense mechanisms against salt stress, which can be broadly classified into avoidance and tolerance strategies. Only some of these strategies are presented below. Salt stress inhibits photosynthesis by reducing water potential. Therefore, the main aim of salt tolerance is to increase water-use efficiency under high salinity (Parida & Das, 2005). There are three distinct types of plant adaptations to salinity: osmotic stress tolerance, Na^+^ or Cl^−^ exclusion, and the tolerance of tissues to accumulated Na^+^ or Cl^−^ (Munns & Tester, 2008). Changes in the composition and structure of cell membranes is a tree defense strategy against salt stress (Milewska-Hendel et al., 2017). The blocking of Cl and Na uptake and redistribution of elements from leaves to other parts of plants (Shelke et al., 2019) and the sequestration of Na^+^ and Cl^−^ into the vacuole (Baetz et al., 2016) can also be effective defense mechanisms. The induction of plant hormones related to ectomycorrhizal fungi may increase salinity tolerance, but it is also possible that there is no such relationship (Zwiazek et al., 2019).

Polyprenols, which are types of polyisoprenoid lipids, play a role in the acclimation of plants to abiotic stress (Bajda et al., 2009). Polyisoprenoid alcohols generally modulate the biophysical properties of biological membranes (Thelin et al., 1995; Skorupinska-Tudek, Wojcik & Swiezewska, 2008) by increasing membrane permeability. Thus, polyprenols help plants adapt to stress conditions (Upchurch, 2008). Trees growing under salt stress have developed defensive methods (Milewska-Hendel et al., 2017): (i) increasing the synthesis of prenyl lipids, acting as scavengers of reactive oxygen species and increasing concentration of Cl^−^ and Na^+^ in leaves, and (ii) biochemical changes in cell walls.

*Acer* species are routinely used in urban plantings in temperate environments, where the trees used are usually limited to a few traditional species. *Acer* species are some of the most common urban trees in Europe and North America (McPherson et al., 1997; Raupp, Cumming & Raupp, 2006; Sjöman, Östberg & Bühler, 2012). Information concerning the adaptation of *A. platanoides* and *A. campestre* to urban environments incorporates the difference between these species (Scharenbroch, 2012). *A. platanoides* has shown high sensitivity to urban environment, mainly to soil salinity in Warsaw, Poland. In 1973–2007, 52,4% of *A. platanoides* trees died and were removed (Dmuchowski, Baczewska & Bra̧goszewska, 2011a). In comparison to *A. platanoides*, *A. campestre*, in addition to being less sensitive, has a greater ability to accumulate dust pollution on leaves (Sæbø, Benedikz & Randrup, 2003).

The aim of this research was to identify the causes of the differences in sensitivity of two *Acer* species (*A. platanoides* and *A. campestre*) to street conditions, especially salt stress, in an urban environment. This paper describes the role of prenols in mitigating salt stress. We assumed that polyprenols could play an important mitigating role. Two types of experiments were performed. First, mature trees were examined as part of field experiments at three locations: main streets in the city centre, city parks (without de-icing) and the suburbs as a control. Second, young trees were tested in a controlled pot experiment with increasing salt doses. The effects of stress in both experiments were examined on the basis of leaf damage.

This research on young and mature trees will determine whether the salt-stress resistance of the species is an innate characteristic or whether it was acquired by acclimation. The pot tests will eliminate factors other than salinity that affect trees growing in urban street environments, such as air pollution, soil pollution (other than salinity), lack of moisture, and soil compaction. The influence of salt stress on plants has been studied for many years. Nevertheless, our knowledge is still insufficient. We hope that our research will significantly expand the knowledge in this area.

## Materials & Methods

The research objects were trees of two *Acer* species that differ in their sensitivity to street conditions within cities: the sensitive Norway maple (*Acer platanoides* L*.*) and the relatively resistant - Field maple (*Acer campestre* L.). Both species belong to the section *Platanoidea Pax*. *A. platanoides* is one of the most frequently planted species in Warsaw. In 1973 and 2002, it accounted for 26% and 22%, respectively, of the trees grown on the main streets of the centre of Warsaw (Dmuchowski, Baczewska & Bra̧goszewska, 2011a). *A. campestre* has so far been rare; however, recently thanks to our recommendations among others, it is planted more often on streets. Basic information on the field and pot experiment methodology is presented in Table 1.

### Study area –field experiment

The climate of Warsaw is moderately continental, characterized by cold winters, with temperatures often below freezing (0 °C), and mild or pleasantly warm summers (Kuchcik et al., 2014). In winter, snowfall is frequent but generally not abundant. Sometimes, cold waves from Siberia can occur, and the temperatures can drop to −20 °C. In summer, there can be short heat waves, with maximum temperatures exceeding 30 °C, and during the hottest periods, reaching as high as 36 °C. The average temperature of the coldest month (January) is −1.8 °C and that of the warmest month (July) is 19.1 °C. However, it must be noted that the amount of salt applied in the street is not determined by the largest negative temperatures but by the frequency of the temperature dropping below 0 °C.

**Figure 1:**
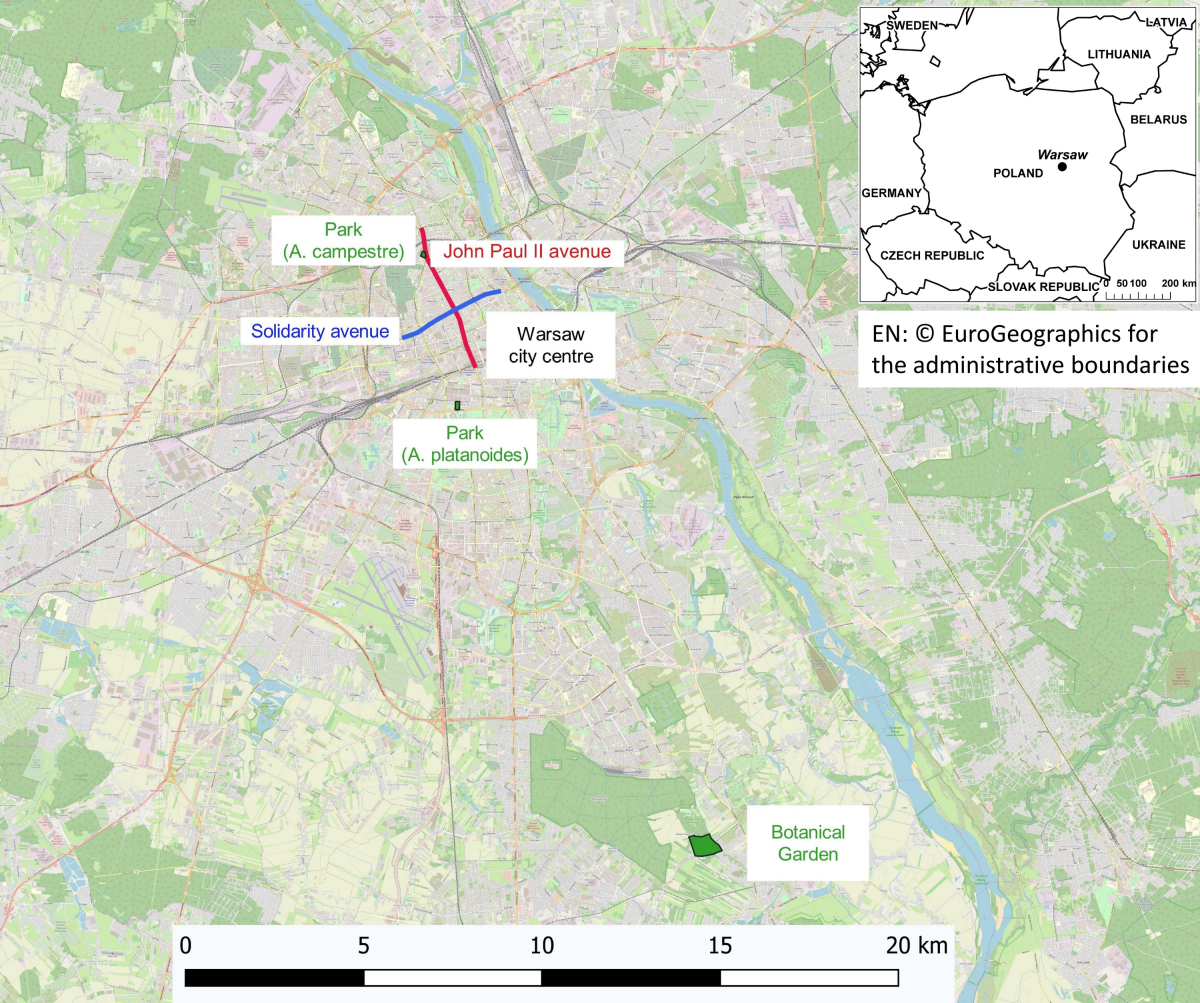
Map showing the areas where the growing clones have been studied. EN:©EuroGeographics for the administrative boundaries.

The study was carried out in three areas of Warsaw (Fig. 1):

a) The street areas are located in the city centre along the main streets with very high- traffic (approximately 60,000 vehicles day), *A. platanoides* trees are present on Solidarity Street and *A. campestre* trees are present on John Paul II avenue, located at a distance of 2 km from each other. During winter, the streets are heavily treated with NaCl for de-icing. In Warsaw, only NaCl is used for de-icing streets and sidewalks. The same compound was used in the pot experiment. The soils in both study areas are anthropogenic and alkaline, and high concentrations of Cl and exchangeable Na, Ca, K and Na (Dmuchowski, Brogowski & Baczewska, 2011b). Warsaw was nearly completely destroyed during World War II; therefore, the soil contains large amounts of building rubble. This fact combined with the concrete slabs covering the areas surrounding the trees made it impossible to collect a representative soil sample. Chemical analysis of leaves provides reliable information about the nutrient conditions and the threat of toxic elements. The nonlinearity of soil-to-plant transfer has been previously reported (Wyttenbach et al., 1998; Chojnacka et al., 2005; Han et al., 2011). The street trees are growing in areas covered with pavement and are 1.5 m from the street. The space occupied by one tree, separated from the next tree by pavement, was only 1.5 m^2^.
b) The park area is a green area located in the city centre in the vicinity of both studied street locations. The trees are growing in a lawn at a distance of 100 m from the street. The soil of the park and the control area was developed to a depth of 50 cm from light silty loam underlined by silt.
c) The control trees are located at the Botanical Garden of the Polish Academy of Sciences in Powsin-Warsaw. This area is an agro-forest area on the southern outskirts of Warsaw, far from local emissions sources and traffic routes.

Paulina Brągoszewska, informed the City Office, Environmental Protection Departament about our research. The person she contacted was Iwona Jędrychowska- Klimczak the then Head of Section for Nature Protection.

**Table 1:** Basic methodological information about the conditions of the experiments with *A. platanoides* and *A. campestre* for the field experiment and the pot experiment with young trees treated with different amounts of NaCl.

	**Field experiment**	**Pot experiment**
Number of trees	2 × 8	6 × 8
Age of trees	∼30	5
Date of salt addition	winter	March
Date of sample collection	middle of July	middle of July
Number of leaves collected from single tree	120	all (∼100)
Trunk diameter		
*A. platanoides*	∼40	3–3.5 cm
*A. campestre*	∼30	3–3.5 cm

### Sampling

Leaves of eight 30-year-old trees of the two studied species were collected at the three locations in mid-July. The tree age was obtained from planting data. Mid-July is recommended for collecting leaves for chemical analysis, because at a later dates, substantially increased amounts of damage will distort the results of these analyses (due to the conversion to a dry matter). Forty leaves were collected from each of the four sides of the trees.

### Pot experiment

The field tests were supplemented with experiments carried out on the same species under controlled conditions in pots. The pot experiment was located in the Botanical Garden of the Polish Academy of Sciences (i.e., the same place as the control area for the field experiment).

The assessment of the influence of soil salinity on the content of biogenic elements and the degree of accumulation of prenyl lipids in the leaves was performed using the plants grown in the pot experiment. Five-year-old *A. platanoides* and *A. campestre* trees were planted in 10-liter pots set on solid ground in separate plots located in the Botanical Garden of the Polish Academy of Sciences.

A detailed description of the pot experiment is presented as previously described in Dmuchowski et al. (2019). The soil used in the experiment was collected from the 0–30 cm soil layer and had a granulometric composition with loamy sand, and the result of the acid reaction test (pH in KCI 1 mol dm-3) was 5.8. The contents of organic carbon and total nitrogen in the soil were 6.12 and 0.63 g kg^−1^, respectively. This soil was developed to a depth of 50 cm and comprised light silty loam underlain by silt. In March, the trees were watered with water that contained dissolved NaCl. Four doses of salt (10, 20, 30, and 40 g) were each dissolved in 0.5 L of water. The control consisted of plants growing in unpolluted pots. The pots with experimental trees were placed outside and watered by rainfall only, similar to the trees in the field experiment. The soil in the pots was covered with ground bark to prevent heating and rapid evaporation of water. The wide range of salt added to the pots was based on the concentrations found in street soils of cities in Poland (Czerniawska-Kusza, Kusza & Duzynski, 2004; Oleksyn et al., 2007; Dmuchowski, Brogowski & Baczewska, 2011b). After 4 months, in mid-July, all the leaves were collected, and the experiment was removed.

### Trees health status

The health condition of trees was assessed on the basis of the degree of leaf damage. This classification consists of six health categories (the leaf damage index), where 0 meant a healthy tree and 5 is a seriously damaged one (damage of up to 75% of the leaf surface area). The same method of leaf health assessment was used in both field and pot experiments. These observations of the health were conducted in mid-July and in the field experiment also assessed in mid-September, because the pot experiment was completed in July.

### Elemental analyses

In both experiments, leaf samples were collected from 8 trees from each species. All the analyses were performed for each tree separately, and the leaf samples were not mixed. The ratio of organic acids to the sum of the anions is considered a good indicator of ion balance in plants (De Wit, Dijkshoorn & Noggle, 1963; Dmuchowski, Brogowski & Baczewska, 2011b; Devitt et al., 2014) therefore, the leaves were not washed after collection because some elements, such as potassium and, to a lesser degree, sodium and chlorine, could be washed away, thus distorting the results. The leaves from the field and pot experiments were collected, and the twigs were removed. The leaves were placed in linen bags, brought to the laboratory and dried for 12 h at 70 °C. The dried material was subsequently ground to powder with an impactor mill (Fritsh14702).

A detailed description of the methodology of chemical analyzes was presented earlier in the paper by Dmuchowski et al. (2019). The powdered samples were dry-mineralized in a muffle oven (Naberthern L40/11/P320, Germany) using the following time/temperature procedure: 120 °C–2 h, 200 °C–1 h, 300 °C–1 h, and 450 °C–5 h (Allen et al., 1974). The ashes were digested in 30% HCl (Merck suprapur) and filtered through a filter paper. Metals (Ca, K, Mg, and Na) and P were determined by atomic spectrophotometry using a Perkin Elmer 1100B (Perkin Elmer, Germany). The weight of the leaf sample was 2 g. Cl was determined by potentiometric titration using an ion-selective electrode and ion metre, Orion Star Plus (Thermo Scientific, USA) (LaCroix, Keeney & Walsh, 1970). S was determined using a LECO-132 elemental analyser (Leco Corporation, USA). N was determined by the Kjeldahl method using a Foss Tecator (Foss Polska, Poland). When determining N, Cl, and S, the sample weight was 0.5 g.

The content of organic acids was calculated in accordance with the method proposed by Van Tuil, Lampe & Dijkshoorn (1964) as the difference between the sum of the cations and the sum of the anions expressed in meq/100 g of dry weight of leaves according to the formula:

\begin{eqnarray*}({\sum }_{\mathrm{K}}-{\sum }_{\mathrm{A}})=R-\mathrm{CO}{O}^{-} \end{eqnarray*}

\begin{eqnarray*}\mathrm{sum~ of~ cations}{\sum }_{\mathrm{K}}={\mathrm{Ca}}^{2+}+{\mathrm{Mg}}^{2+}+{K}^{+}+{\mathrm{Na}}^{+} \end{eqnarray*}

\begin{eqnarray*}\mathrm{sum~ of~ anions}{\sum }_{\mathrm{A}}={H}_{2}{\mathrm{PO}}_{4}^{-}+{\mathrm{SO}}_{4}^{2-}+{\mathrm{Cl}}^{-}. \end{eqnarray*}

The N content was omitted in the calculations due to the presence of traces of nitrates (NO_3_^−^) in the leaves.

For quality control (QC), the elemental content in the plant samples was determined by certified reference materials, including apple leaves (1515) from the National Institute of Standards and Technology (USA) and beech leaves (ERM100) from European Reference Materials. The results of the chemical analyzes were in agreement with the certified values. The range of recovery calculated on the basis of comparing the results of the measured content of elements in the certified material with the declared contents was 95.9%–106.2% for all elements (Table 2).

**Table 2:** Comparisons of measured and certified concentrations of elements in certified materials): Apple Leaves 1515 (National Institute of Standard and Technology, USA).

**Element**	**Certified**	**Measured**	**Recovery (%)**
Cl mg g^−1^	579 ± 23	601 ± 25	103.8
Na mg g^−1^	24.4 ± 1.2	23.4 ± 1.1	95.9
N (%)	2.25 ± 0.19	2.29 ± 0.21	101.8
P (%)	0.16 ± 0.011	0.17 ± 0.01	106.2
K (%)	1.61 ± 0.02	1.56 ± 0.05	96.9
Ca (%)	1.57 ± 0.015	1.65 ± 0.05	105.1
Mg (%)	0,27 ± 0.008	0.28 ± 0.01	103.7
S (%)	0.18	0.19 ± 0.01	105.07

### Qualitative and quantitative analysis of polyprenols

The internal standard (Prenol-15, 50 µl, *C* = 1 µg/ µl) with 1 ml of an acetone: methanol mixture (1:1, v/v) were added to dried and ground leaves (50 mg), and then incubated at 37 °C for 37 min. After centrifugation and decantation, the extract was obtained. The pellets were re-extracted in 1 ml of aceton-hexane mixture (1:1), in an ultrasound bath and centrifuged again. This procedure was repeated 4 times. All extracts were pooled and evaporated under a stream of nitrogen. The dry residue was subjected to the process of hydrolysis according with the procedure proposed by Skorupinska-Tudek, Wojcik & Swiezewska (2008). After hydrolysis, the lipids were purified as described earlier by Skorupinska-Tudek, Wojcik & Swiezewska (2008). The lipids were isolated by column chromatography on silica gel in hexane. After the application of the sample a step gradient was applied: 2% diethyl ether in hexane, followed by 15% diethyl ether in hexane (10 ml). The latter fraction was evaporated in a nitrogen stream, dissolved in 200 µl of IPA - EtOH mixture. Polyprenols were identified by HPLC /UV method. Quantitative and qualitative analyses were performed. The HPLC/UV conditions were consistent with the previously described methodologies of Skorupinska-Tudek, Wojcik & Swiezewska (2008) and Jozwiak et al. (2013). A linear gradients of solvent mixtures A (90% methanol in water, v/v) and B (50% methanol, 25% hexane and 25% isopropanol v/v/v) at a flow of 1.5 ml/min was used during analyses, which lasted 25 min. The polyprenols were identified by comparing their retention times and absorption spectra with the corresponding parameters of standard substances (using an external standard). Separated compounds were identified on the basis of comparison of their retention time and absorption spectrum with appropriate parameters of external standard polyprenol mixture (Collection of Polyprenols from the Institute of Biochemistry and Biophysics, Polish Academy of Sciences Warsaw, Poland). The results were integrated using the Empower Pro program.

The content of polyprenols was estimated based on the signal of the internal standard (Pren-15), and the calculated values were given as mg per 1 gram of dry leaves.

The results are the mean of three independent analyses. To provide quality control, additional analyses were performed on plant material with a well-characterized polyprenol contents and spectra (photosynthetic tissue of *Sorbus intermedia*, *Nicotiana tabacum* and *Picea abies*).

### Statistical analysis

Comparisons of means were performed using one-way ANOVA, and homogenous groups of means were distinguished using Tukey’s HSD post-hoc procedure at 0.05 significance level. Relationships between variables were evaluated using Spearman’s correlation coefficients. Principal component analysis (PCA) was used for multivariate relationships for studied variables and multivariate differences between the objects. The analyses were performed in Statistica 13 program.

## Results

### Field experiment

**Figure 2:**
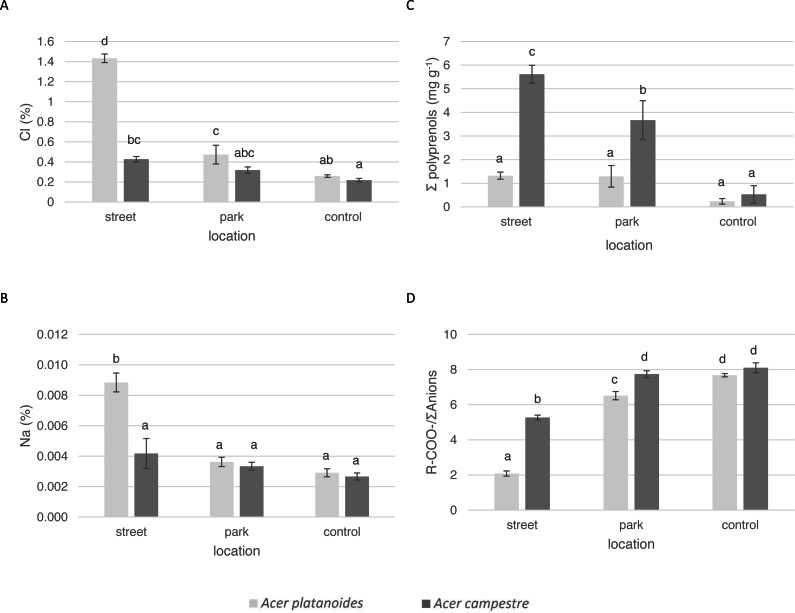
Influence of the growth site (street, park, and control) on the content of macronutrients in the leaves of *A. platanoides* and *A. campestre* in the field experiment: (A) Cl content; (B) Na content; (C) polyprenol content; and (D) ratio of organic acids to the sum of anions. Different letters indicate significant differences between the sites and species (*P* < 0.05). Error bars present standard errors (SE).

The data from the evaluation of the tree health and the elemental content and the ion balance indicators of the leaves of trees treated with de-icing salt from the city centre, park and control areas are presented in Table 3 and Fig. 2. Tree health was assessed on the basis of the health of the leaves. The *A. campestre* leaves were in good condition, and no damage was observed at any of the three locations on either observation date. In the park and the control area, the *A. platanoides* leaves presented no damage on either date. However, there was marginal necrosis, which is a characteristic of salt stress, on the leaves of the trees growing along the streets. The average leaf damage index was estimated in mid-July and in mid-September, yielding values of 1.63 and 3.88, respectively.

**Table 3:** Content of macronutrients and S, means and SDs (*n* = 8) and leaf damage index for *A. platanoides* and *A. campestre* leaves growing at the different sites (street, park, and control) and *P*-values based on ANOVA. Lack of the same letter next to the means indicate significant differences between the means for species *x* locations (for all six means in columns) *P*-values below 0.05 indicate a significant effect of species or location or the interaction of species × location.

	**Leaf damage index - July**		**Leaf damage index - September**		**N**		**S**		**P**		**K**			**Mg**		**Ca**	
	**Mean**	**SD**	**Mean**	**SD**	**%**	**SD**	**%**	**SD**	**%**	**SD**	**%**	**SD**	**%**		**SD**	**%**	**SD**
A. ***platanoides***																	
**street**	1.63^b^	0.74	3.88^b^	0.83	2.12^a^	0.13	0.19^a^	0.01	0.22^a^	0.06	1.25^b^	0.13	0.15^a^		0.06	1.66^a^	0.24
**park**	0.00^a^	0.00	0.00^a^	0.00	2.53^b^	0.19	0.22^b^	0.03	0.19^a^	0.06	1.06 a	0.13	0.20^ab^		0.03	1.63^a^	0.21
**control**	0.00^a^	0.00	0.00^a^	0.00	1.91^a^	0.30	0.18^a^	0.03	0.31^b^	0.04	1.25 b	0.17	0.23^b^		0.04	1.45^a^	0.18
A. ***campestre***																	
**street**	0.00^a∗^	0.00	0.00^a∗^	0.00	1.68^a∗^	0.16	0.17^a^	0.01	0.16^a∗^	0.02	0.91 a*	0.13	0.12^a^		0.02	1.43^a∗^	0.16
**park**	0.00^a^	0.00	0.00^a^	0.00	2.49^b^	0.19	0.21^b^	0.02	0.17^a^	0.02	1.11 b	0.10	0.21^b^		0.04	1.65^b^	0.19
**control**	0.00^a^	0.00	0.00^a^	0.00	2.36^b∗^	0.14	0.20^b^	0.01	0.32^b^	0.10	1.14 b	0.26	0.23^b^		0.07	1.39^a^	0.22
**P-values based on ANOVA**																	
Species (S)	<0.001		<0.001		0.878		0.531		0.147		0.006		0.849			0.126	
Location (L)	<0.001		<0.001		<0.001		<0.001		<0.001		0.099		<0.001			0.013	
Interaction S × L	<0.001		<0.001		<0.001		0.018		0.222		0.005		0.500			0.204	

The average Cl content in the leaves of *A. platanoides* on the street was 1.43% (Fig. 2) and was significantly greater than the Cl content in the leaves of *A. campestre* trees (0.43%). There was significantly less Cl in the leaves of park trees for both species. The Na content in the leaves of *A. campestre* did not differ significantly among the three locations. However, the *A. platanoides* street trees leaves contained 0.009% Na, which is significantly greater than that of the *A. platanoides* leaves from other sites and greater than that of the *A. campestre* street tree leaves (0.004%). In the case of macronutrient content, the relationships between species and locations were not as clear as those in the cases of Cl and Na (Table 3; Fig. 2). The leaves of *A. platanoides* street trees contained significantly more N, P, K and Ca than the leaves of *A. campestre*, but the differences were not statistically significant. The ion balance indicator of the leaves of both species was greatest for the control trees (8.10 in *A. campestre* and 7.68 in *A. platanoides*), relatively low for the park trees, and lowest for the street trees (5.27 and 2.08, respectively). The differences in the indicator values between the street and park trees were significant for both species.

In the leaves of both *Acer* species in both experiments, four polyprenols were identified: Pren-10, Pren-11, Pren-12 and Pren-13. In both experiments, only the total sum of the polyprenol content was assessed. There were large differences in the total amount of polyprenols in the leaves of the tested *Acer* species. In urban *A. campestre*, the total content of polyprenols (street trees, 5.62 mg g^−1^, and park trees, 3.67 mg g^−1^) was significantly higher than that in *A. platanoides* (street trees, 1.32 mg g^−1^, and park trees, 1.29 mg g^−1^). In the control trees, these values were significantly lower (*A. campestre* - 0.53 mg g^−1^, and *A. platanoides* - 0.23 mg g^−1^) and did not differ significantly between the two species (Fig. 2).

**Figure 3:**
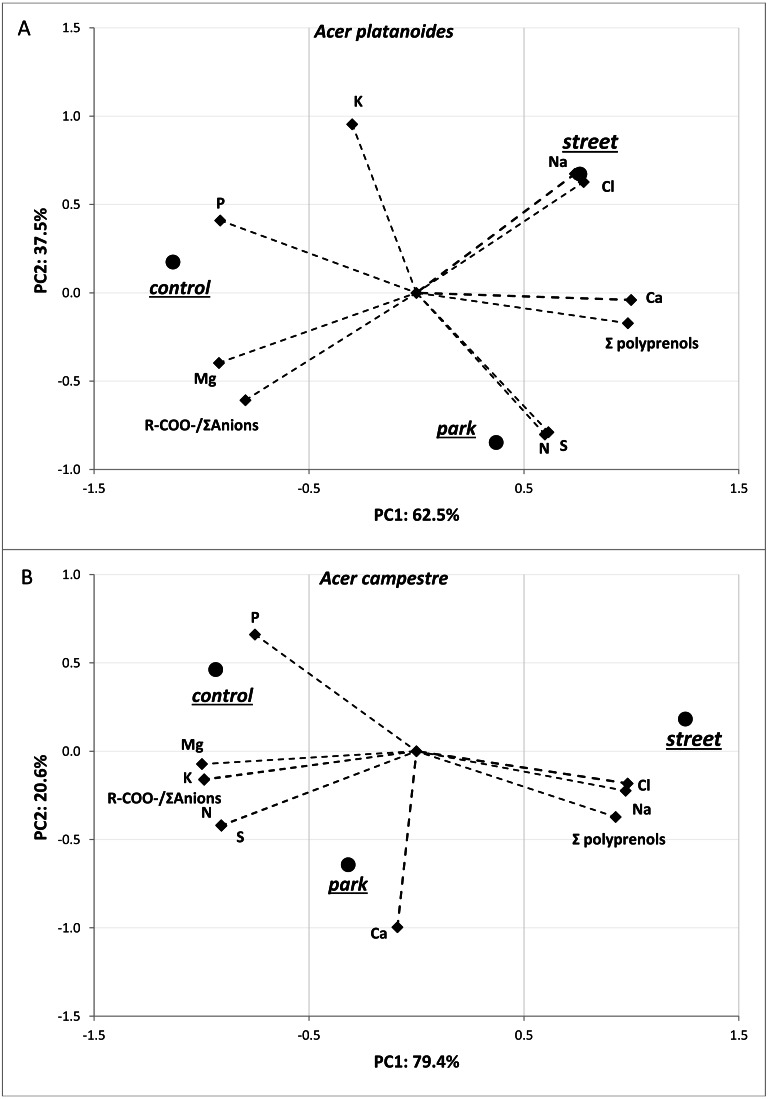
Results of PCA for the field experiment presenting two principal components (A: PC1 and B: PC2) for the different sites and loadings for the variables included in the analysis.

The results of the principal component analysis (PCA) presented in Fig. 3 allow us to evaluate multivariate relationships between the variables and to characterize the multivariate differences between trees located in different places. For both tree species, a very strong positive relationship was detected between Na and Cl. Moreover, these two elements were positively correlated with total polyprenols, and stronger relationships were observed for *A. campestre* than for *A. platanoides*. High Na and Cl contents and a high total polyprenols content were detected in the street trees, while the lowest contents were found in the control trees. The largest contents of Mg, P and R-COO-/Σanions value were found in the control trees of both species. The trees located in the park had moderate amounts of most elements. In the case of *A. platanoides*, high N and S contents were detected in the trees located in the park, while in the case of *A. campestre*, a high Ca content was found. Most of the relationships between the element contents were similar between the two trees species.

### Pot experiment

In the pot experiments, the leaves of *A. campestre* showed no damage for any of the trees in the experiment. In the case of *A. platanoides*, damage occurred in response to 20 mg L^-1^ NaCl (damage index 0.63), and the damage index increased to 3.00 in response to the highest dose (40 mg^L-1^).

**Figure 4:**
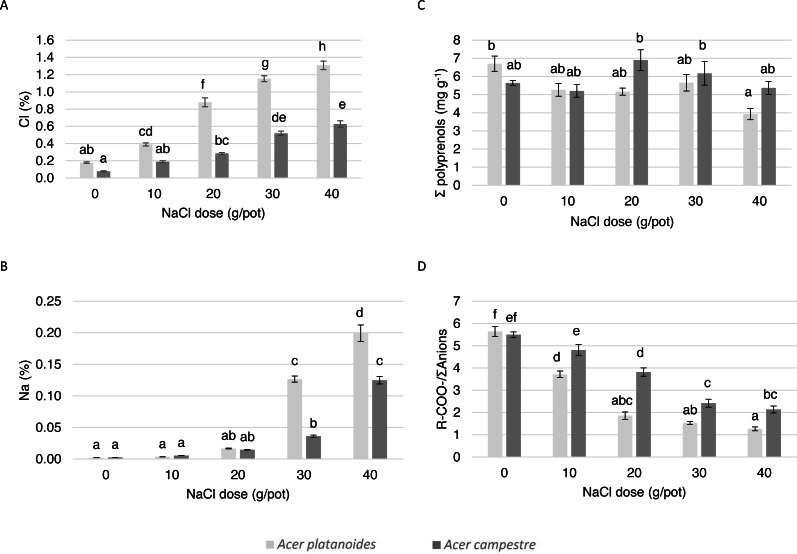
The effect of increasing doses of NaCl on the chemical composition of leaves (*n* = 8) of *A. platanoides* and *A. campestre* in the pot experiment: (A) Cl content, (B) Na content, (C) polyprenol content, and (D) ratio of organic acids to the sum of anions. Different letters indicate significant differences between the doses and species (*P* < 0.05). Error bars present standard errors (SE).

The elemental composition and ionic balance of the indicators of the leaves from the trees treated with increasing NaCl doses in the pot experiment are presented in Table 4 and Fig. 4. The Cl content in the leaves of both species increased with increasing NaCl dose. Compared with the leaves of *A. campestre*, the leaves of *A. platanoides* accumulated more Cl, and the differences in accumulation between the species were statistically significant for all NaCl doses. A similar tendency was detected for the Na content in the leaves.

The analysis of the contents of macronutrients and S in the leaves of both *Acer* species did not result in a clear trend. In general, increasing the NaCl dose applied to the pots did not have a significant effect on the N, S, P or Mg contents. Differences in the S, P or Mg contents did not occur between the species. The *A. platanoides* leaves contained significantly more N, K, and Ca than that the *A. campestre* leaves at the same NaCl doses.

The value of the ion balance indicator of the leaves significantly decreased with increasing doses of NaCl. In *A. platanoides*, the indicator value decreased from 5.64 in the control leaves to 1.27 in response to the largest NaCl dose (40 mg L^−1^); in *A. campestre,* the value decreased from 5.50 to 2.14. In the control trees, there were no differences between the species.

The effect of increasing doses of NaCl on the content of polyprenols in the leaves of both species did not differ. In general, compared with the *A. platanoides* leaves, the *A. campestre* leaves presented more polyprenols, although this effect was more pronounced at NaCl concentrations greater than 20 mg L^−1^.

The Spearman correlation coefficients (Table 5) between the NaCl dose and the content of macronutrients, the total polyprenols content and the ion balance indicators suggested that the level of soil salinity was positively correlated with the Cl, Na and K content in the leaves of both *Acer* species and negatively correlated with the ion balance index. In *A. platanoides*, there was also a negative correlation between soil salinity and the polyprenol content.

**Table 4:** Means and SDs for the content of macronutrients, S and the leaf damage index for *A. platanoides* and *A. campestre* leaves from the pot experiment (number of replicates *n* = 8) and *P*-values based on ANOVA. The trees grew under increasing NaCl doses. ack of the same letter next to the means indicate significant differences between the means for the doses of NaCl ×species (for all ten means in columns) *P*-values below 0.05 indicate a significant effect of species or dose or the interaction of species ×dose.

**dose**	**Leaf damage index**		**N (%)**		**S (%)**		**P (%)**		**K (%)**		**Mg (%)**		**Ca (%)**	
**NaCl**	**Mean**	**SD**	**Mean**		**SD**	**Mean**		**SD**	**Mean**		**SD**	**Mean**		**SD**	**Mean**		**SD**	**Mean**		**SD**	
***A. platanoides***														
**0**	0.00^a^	0.00	1.60^a^		0.06	0.17^a^		0.01	0.26^a^		0.02	0.92^a∗^		0.07	0.17^a^		0.03	0.72^a∗^		0.09	
**10**	0.00^a^	0.00	1.48^a∗^		0.19	0.16^a^		0.02	0.26^a^		0.04	0.97^a∗^		0.16	0.17^a^		0.03	0.77^a∗^		0.13	
**20**	0.63^b∗^	0.52	1.55^a∗^		0.15	0.16^a^		0.02	0.31^b^		0.04	1.05^ab∗^		0.14	0.19^a^		0.02	0.80^a∗^		0.10	
**30**	1.63^c∗^	0.74	1.62^a^		0.22	0.17^a^		0.02	0.29^ab^		0.04	1.20^bc∗^		0.23	0.18^a^		0.03	0.80^a∗^		0.13	
**40**	3.00^d∗^	0.53	1.56^a∗^		0.12	0.16^a∗^		0.01	0.26^a^		0.03	1.24^c∗^		0.15	0.18^a^		0.03	0.71^a∗^		0.09	
***A . campestre***														
**0**	0.00^a^	0.00	1.56^a^		0.22	0.16^a^		0.01	0.27^a^		0.03	0.65 a		0.14	0.16^a^		0.02	0.50^a^		0.06	
**10**	0.00^a^	0.00	1.77^ab^		0.34	0.17^ab^		0.01	0.29^a^		0.03	0.66 a		0.08	0.18^a^		0.02	0.69^c^		0.13	
**20**	0.00^a^	0.00	1.85^b^		0.21	0.18^b^		0.01	0.29^a^		0.06	0.73 a		0.10	0.19^a^		0.03	0.69^c^		0.14	
**30**	0.00^a^	0.00	1.82^ab^		0.29	0.17^ab^		0.01	0.28^a^		0.04	0.87 b		0.12	0.17^a^		0.03	0.61^bc^		0.09	
**40**	0.00^a^	0.00	1.71^ab^		0.27	0.17^b^		0.02	0.28^a^		0.03	0.91 b		0.14	0.19^a^		0.03	0.57^ab^		0.11	
***P*-values based on ANOVA**														
**Species (S)**	-		0.001		0.169		0.451		<0.001		0.819		<0.001	
**Dose (D)**	-		0.368		0.847		0.139		<0.001		0.181		0.002	
**Interaction (S × D)**	-		0.187		0.053		0.293		0.974		0.757		0.337	

**Figure 5:**
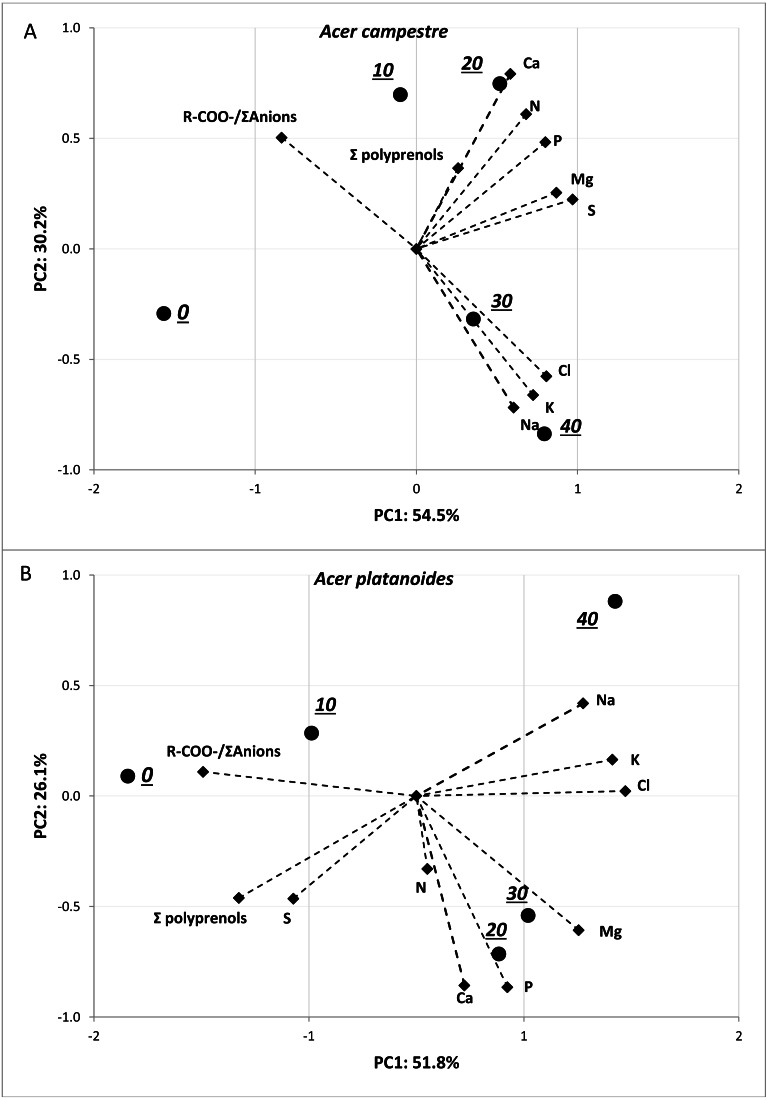
Results of PCA for the pot experiment presenting two principal components (A: PC1 and B: PC2) for the different doses of NaCl and loadings for the variables included in the analysis.

The results of the PCA presented in Fig. 5 allowed us to evaluate the multivariate relationships between the variables (i.e., macronutrients content, ion balance index and total polyprenols) to characterize the multivariate differences between the treatments with various doses of NaCl. The analyses were performed separately for each tree species. For both tree species, strong positive relationships were observed among the Na, Cl and K contents. A negative correlation between these three elements and R-COO-/Σanions was detected. The greatest contents of Na, Cl and K were detected in response to doses of 40 mg L^−1^NaCl, and the lowest were detected in response to doses of 0 and 10 mg L^−1^ NaCl for both tree species. In the case of *A. platanoides*, the total polyprenols content was strongly negatively correlated with Na, Cl and K content, while in the case of *A. campestre*, this correlation was weak.

## Discussion

In this study, evaluations of the health of the two *Acer* species based on leaf assessment confirmed the differences between the two species. According to the literature (see Introduction), and the results from our field and pot experiments, *A. platanoides* is much more sensitive than *A. campestre* to urban street conditions, especially soil salinity.

Differences in the health conditions of the street trees of both species were accompanied by differences in the contents of Cl and Na in the leaves. The *A. platanoides* leaves contained significantly more Cl than *A. campestre* leaves; the Cl content in the *A. platanoides* leaves was more than 3 times greater than that in the *A. campestre* leaves in the trees growing under comparable street conditions and in the pot experiments at all salinity levels. However, the differences in the Na content between the tested species were relatively small. The *A. platanoides* street tree leaves contained approximately twice as much as the *A. campestre* street tree leaves. In the pot experiment, significant differences occurred under relatively high levels of salinity. A comparison of these two *Acer* species with other species was not needed because of the very large variation between species in transporting Cl and Na to leaves and because the stress associated with salt blocks this transport mechanism (Tattini et al., 1994; Alaoui-Sosse et al., 1998). The content of Cl in the leaves of street trees in Warsaw growing under comparable conditions ranged from 0.04% in *Quercus rubra* and 0.27% in *Gleditsia triacanthos* to 1.88% in *Tilia* x*euchlora*, and the Na levels ranged from 0.001 in *Quercus rubra* and *Robinia pseudoacacia* to as high 0.329% in *T.* x*euchlora* (Dmuchowski et al., 2013).

**Table 5:** Correlation coefficients (*P*-values in brackets) between dose of NaCl and content of macronutrients, S, polyprenol content and ratio of organic acids to the sum of anions.

	***A. platanoides***	***A. campestre***
	**NaCl dose**	**NaCl dose**
Cl	0.956 (<0.001)	0.959 (<0.001)
Na	0.977 (<0.001)	0.980 (<0.001)
N	0.008 (0.959)	0.227 (0.159)
S	−0.184 (0.256)	0.358 (0.023)
P	0.087 (0.595)	0.086 (0.599)
K	0.577 (<0.001)	0.663 (<0.001)
Mg	0.090 (0.579)	0.209 (0.195)
Ca	0.012 (0.940)	0.126 (0.437)
R-COO-/ ΣAnions	−0.908 (<0.001)	−0.913 (<0.001)
Sum of polyprenols	−0.557 (<0.001)	<0.001 (1.000)

The extent of tree injury and mortality is correlated with leaf Cl and/or Na concentration (Calvo-Polanco, Zwiazek & Voicu, 2008; Goodrich & Jacobi, 2012; Equiza et al., 2017). By testing Cl^−^ and Na^+^ ions separately, Genc et al. (2015) showed that Na affects plants mainly osmotically and that Cl affects plants osmotically and is toxic to plants. Chlorine ions are considered more toxic than sodium ions to tress. The Cl content of leaves is more strongly correlated than the Na content with the degree of leaf damage (Alaoui-Sosse et al., 1998; Paludan-Müller et al., 2002; Kayama et al., 2003). Munns & Tester (2008) found that in the case of trees, sodium ions are retained in the roots and shoots, and only part of the charge reaches the leaves. Na toxicity does not manifest as such but rather as deficiencies of other exchangeable cations (Ca, K and Mg) because of the competition from Na for uptake; leaf damage is mainly caused by Cl (Slabu et al., 2009a; Slabu et al., 2009b; Genc et al., 2015). However, there are publications in the literature proving that Na is more toxic to plants than Cl (Tester & Davenport, 2003). In the present study, the chemical analyses revealed very high variability in the Na content in the leaves of both *Acer* species with respect to the street trees and in the pot experiment. Despite having the same leaf damage index value, the range of the Na content was very wide. The variation in the content of Cl was much smaller. The results obtained from both experiments suggest a greater effect of Cl than Na on the health of *Acer* under high soil salinity conditions. The same conclusions were drawn from the research of Baczewska et al. (2014) and Genc et al. (2015). However, our own research and literature review do not allow us to draw a final conclusion on this matter.

The content of macronutrients (N, P, K, and Mg) in the leaves of both species was considered as normal, according to Kopinga & Van den Burg (1995). A deficiency in content was not observed in any variant in either experiment. The analysis of the content of macronutrients and S in both species of *Acer* and in both experiments revealed no significant trends. A lack of a significant impact of soil salinity on the content of macronutrients was confirmed by Marosz & Nowak (2008). Significant differences between experiment variants were due to different conditions between field and pot experiment. The reason for the higher Ca content in street trees is soil contamination with building rubble containing high levels of calcium compounds, dating back to World War II (see the Study area section). The N contents were higher in the leaves of street trees from the city centre, than in the leaves of the trees in the pot experiments or from the suburbs, this is probably caused by NOx air pollution in the city centre. Similar results were found in other publications discussing research from Warsaw (Dmuchowski, Brogowski & Baczewska, 2011b).

Disruption of ion balance caused by salt stress affect plant growth and development (e.g., Mazher, El-Quesni & Farahat, 2007; Devitt et al., 2014; Dmuchowski et al., 2014). In the majority of related studies, plant elements contents are given in units of weight (e.g., mg kg^−1^ or %), which is over-simplified (De Wit, Dijkshoorn & Noggle, 1963; Van Tuil, Lampe & Dijkshoorn, 1964). This fact substantially limits the possibility of discussing our results in comparison with those of other studies. The ratio of organic acids to the sum of anions in the leaves expressed as milliequivalents is considered a good indicator for characterizing the health of trees. Disturbing the ion balance reduces the value of this indicator (Devitt et al., 2014). In the field experiments in the present study, the value of the ion balance indicator of the leaves of street trees was significantly lower than that in the park and control trees. The value of this indicator was twice as high for the leaves of *A. campestre* that were undamaged, as the leaves of *A. platanoides*, which were significantly damaged (the average leaf damage index in mid-July and mid-September was 1.63 and 3.86, respectively). In the pot experiment, the values of the indicator for both species decreased significantly with increasing NaCl dose, but the indicator value was significantly greater for the *A. campestre* leaves than for the *A. platanoides* leaves at all doses of NaCl.

Four polyprenols were identified in both *Acer* species, as reported previously studies (Chouda & Jankowski, 2005). There were significant differences in leaf polyprenol content between the mature trees in the field experiment and young trees in the pot experiment. In the pot experiment, no clear relationship was found between the polyprenol content and the *Acer* species and NaCl dose used. In the field experiment, compared with the *A. platanoides* leaves, the *A. campestre* leaves from the urban sites (streets and park) contained more polyprenols. There were also more polyprenols in the *A. campestre* leaves from the street trees than from the park trees. The increased polyprenol content in the *A. campestre* street tree leaves were accompanied by a reduction in the Cl and Na content compared with the content in the *A. platanoides* street tree leaves*.* These results suggest that the *A. campestre* strategy for mitigating salt stress may rely on its ability to synthesize polyprenols acquired during acclimation. Only the mature street trees had this ability, and young trees in the pot experiment did not develop it.

Previous studies have also shown that polyprenols are involved in mitigating salt stress. The ability of *G. biloba* to synthesize increased polyprenols under salt stress is similar to that of mature *A. campestre* trees (Dmuchowski et al., 2019). Research on the use of *T. xeuchlora* as street trees under salt stress conditions suggests a protective role of polyprenols in limiting the accumulation of Cl in the leaves, which results in reduced leaf damage. The polyprenol content was highest in the leaves of the “healthy” trees, lower in the “sick” trees, and lowest in the control group (Baczewska et al., 2014). Another study (Milewska-Hendel et al., 2017) on *T. xeuchlora* confirmed that the levels of polyprenols were higher in the leaves from healthy trees than those from other trees; however, the results of the anatomical studies suggest that the differences that were detected in the apoplast and symplasm may be part of the defensive strategy of *T*. *xeuchlora* trees against salt stress, which relies on changes in the chemical composition of the cell wall with respect to the pectic and AGP epitopes and an increased synthesis of polyprenols.

Our research was limited to the studying the effect of salt stress only on the contents of Na and Cl, macronutrients, and polyprenols in the leaves, and as well the ion balance index of the leaves. Many plant strategies related to the mitigation of salt stress have not been considered. These strategies include the following: inducing high antioxidant activity; reducing the content of free radicals and reactive oxygen species (ROS) in leaves (Malik et al., 2011); compartmentalizing Na^+^ and Cl^−^ in the vacuoles (Baetz et al., 2016); inducing biosynthesis of compatible solutes, such as proline, glycine betaine, sugars, and polyols (Parida & Das, 2005); inducing antioxidative enzymes (Sorkheh et al., 2012); and inducing plant hormones (Ryu & Cho, 2015).

## Conclusions

The two experimental methods used in this study (i.e., the field and in pot experiments) allowed for a more complete assessment of tree strategies for mitigating salt stress. *A. campestre* displayed a more specific strategy than did *A. platanoides*. This strategy was based on several elements. *A. campestre* limited Cl and Na transport to the leaves, which resulted in no damage to those organs. Under the same conditions, *A. platanoides* contained more Cl and Na in their leaves and were seriously damaged. The disruption of ion balance caused by salt stress was also lower in *A. campestre* than in *A. platanoides,* as expressed by the higher values of the ionic balance indicator. *A. campestre* synthesized larger amounts of polyprenols, which probably have the ability to mitigate salt stress. This ability was acquired during the process of acclimation because it occurred only in older trees in the field experiment and was not seen in the young trees in the pot experiment. The ability to synthesize lipid polyprenols may be one of the factors that alleviate salt stress, but our research does not allow us to conclude that they are the decisive factor. Salt stress did not affect the occurrence of macronutrient deficiency in the leaves of either species.

Our research confirmed that *A*. *campestre* is suitable for planting along streets in cities under the most unfavourable conditions for trees, especially salt stress. This study also confirmed the high sensitivity of *A. platanoides* to salt stress. Therefore, *A. platanoides* should not be planted along streets with heavy traffic, where large amounts of salt are used in winter for de-icing.

##  Supplemental Information

10.7717/peerj.10577/supp-1 Supplemental Information 1 Raw data.

## Additional Information and Declarations

### Field Study Permissions

The following information was supplied relating to field study approvals (i.e., approving body and any reference numbers): Paulina Brągoszewska, informed the City Office, Environmental Protection Departament about our research. The person she contacted was Iwona Jędrychowska- Klimczak the then Head of Section for Nature Protection.

### Data Availability

The following information was supplied regarding data availability: Raw data are available as a Supplemental File.
